# Dysfunction of the Brown Adipose Organ in HFD-Obese Rats and Effect of Tart Cherry Supplementation

**DOI:** 10.3390/antiox13040388

**Published:** 2024-03-23

**Authors:** Vincenzo Bellitto, Maria Gabriella Gabrielli, Ilenia Martinelli, Proshanta Roy, Giulio Nittari, Paolo Cocci, Francesco Alessandro Palermo, Francesco Amenta, Maria Vittoria Micioni Di Bonaventura, Carlo Cifani, Daniele Tomassoni, Seyed Khosrow Tayebati

**Affiliations:** 1 School of Medicinal Sciences and Health Products, University of Camerino, 62032 Camerino, Italy; vincenzo.bellitto@unicam.it (V.B.); ilenia.martinelli@unicam.it (I.M.); proshanta.roy@unicam.it (P.R.); giulio.nittari@unicam.it (G.N.); francesco.amenta@unicam.it (F.A.); mariavittoria.micioni@unicam.it (M.V.M.D.B.); carlo.cifani@unicam.it (C.C.); 2 School of Biosciences and Veterinary Medicine, University of Camerino, 62032 Camerino, Italy; gabriella.gabrielli@unicam.it (M.G.G.); paolo.cocci@unicam.it (P.C.); francesco.palermo@unicam.it (F.A.P.); daniele.tomassoni@unicam.it (D.T.)

**Keywords:** obesity, brown adipose tissue, antioxidants, high-fat diet, whitening, browning, diet-induced obesity, tart cherry

## Abstract

Obesity has a great impact on adipose tissue biology, based on its function as a master regulator of energy balance. Brown adipose tissue (BAT) undergoes remodeling, and its activity declines in obese subjects due to a whitening process. The anti-obesity properties of fruit extracts have been reported. The effects of tart cherry against oxidative stress, inflammation, and the whitening process in the BAT of obese rats were investigated. Intrascapular BAT (iBAT) alterations and effects of *Prunus cerasus* L. were debated in rats fed for 17 weeks with a high-fat diet (DIO), in DIO supplemented with seed powder (DS), and with seed powder plus the juice (DJS) of tart cherry compared to CHOW rats fed with a normo-caloric diet. iBAT histologic observations revealed a whitening process in DIO rats that was reduced in the DS and DJS groups. A modulation of uncoupling protein-1 (UCP-1) protein and gene expression specifically were detected in the obese phenotype. An upregulation of UCP-1 and related thermogenic genes after tart cherry intake was detected compared to the DIO group. Metabolic adjustment, endoplasmic reticulum stress, protein carbonylation, and the inflammatory microenvironment in the iBAT were reported in DIO rats. The analysis demonstrated an iBAT modulation that tart cherry promoted. In addition to our previous results, these data confirm the protective impact of tart cherry consumption on obesity.

## 1. Introduction

The development and expansion of obesity greatly impact adipose tissue biology, based on its function as a master regulator of energy balance and nutritional homeostasis [1]. When energy intake chronically exceeds energy expenditure, weight gain and obesity result. This excess weight is overstored fat in adipose tissue, mainly localized in the abdominal region [2]. This tissue is not only a passive storage depot but also an endocrine organ that secretes molecules, such as adipokines, that can regulate appetite and metabolism throughout the body. In addition to these energy-storing fat cells, there are also brown fat cells, which obtained their name from their high number of mitochondria and are specialized in releasing energy in the form of heat, a process called non-shivering thermogenesis [3]. The functional properties of brown adipose tissue (BAT) are based on the specific expression of mitochondrial uncoupling protein 1 (UCP-1) and the high number of β-adrenergic receptors that mediate cold-induced lipolysis [3]. It has been reported that targeting the activity of BAT and tanning white adipose tissue (WAT) leads to an increase in energy expenditure. Thus, this is an approach to preventing obesity [4]. Concurrently, β-adrenergic signaling in BAT activates the expression of *peroxisome proliferator-activated receptor γ coactivator 1α (PGC-1α)*, which stimulates the expression of UCP-1 and mitochondrial genes [5].

In addition to an anatomical reinterpretation of adipose tissue, a recent concept has attracted research interest, which is the plasticity of the adipose tissue. Such a property is based on the occurrence of a third type of adipose cells set in WAT but resembling the brown adipocyte phenotype [6]. These cells are referred to as brite (brown-in-white), beige, or paucilocular adipocytes due to their multilocular morphology. The beige cells resemble white fat cells in having an extremely low basal expression of UCP-1, but like classical brown fat, they respond to cyclic AMP stimulation with high UCP-1 expression and respiration rates [7].

The main adipokines released by WAT (e.g., leptin and adiponectin) are poorly expressed in BAT, especially when it is thermogenically active. Indeed, the expression levels of leptin and/or adiponectin are even used as WAT versus BAT markers in situations of adipose tissue plasticity [8]. However, leptin gene expression in brown fat is somewhat controversial because some authors report its minimal expression, whereas others report high expression of it [9]. BAT is composed of numerous cell types, including varying numbers of white adipocytes [10]. This tissue undergoes dramatic remodeling in response to nutritional factors by increasing the size of individual cells (hypertrophy) and recruiting new adipocytes from the resident pool of progenitors (hyperplasia). The hypertrophic growth of beige adipocytes is associated with a relative deficiency of vasculature that creates a local imbalance between oxygen supply and consumption, which in turn leads to increased levels of angiogenic factors and the expression of inflammation and endoplasmic reticulum stress-associated genes [11]. At the cellular level, one of the alterations in hypertrophic adipocytes is the accumulation of radical oxygen species and dysfunction of the endoplasmic reticulum (ER). In accordance, chronic obesity is associated with ER stress in beige adipose tissue, and free fatty acids induce reactive oxygen species (ROS) generation as well as ER stress by activating unfolded protein response (UPR) signaling pathways in adipocytes [12]. Recent studies have identified infiltrated immune cells in BAT and inflammatory processes as contributors to BAT dysfunction in obesity and associated metabolic disorders [13]. However, BAT appears to be more resistant to infiltration by macrophages in diet-induced obese mice than WAT, as these cells only appear after a longer time and have a lesser impact on BAT. Furthermore, the expression of inflammatory markers is lower in BAT than in WAT [13]. BAT mass and activity decline in obese subjects, primarily due to increased lipid deposition leading to the conversion of brown adipocytes to white-like unilocular cells, called BAT whitening. This process is characterized by crown-like structure (CLS) formation, enlarged ER, degenerating mitochondria, and collagen fibrils interspersed in the tissue. Altogether, these findings suggest that converted enlarged brown adipocytes are highly prone to death, which, by promoting inflammation in whitened BAT, may contribute to the typical stress state seen in obesity [14].

Obesity-induced insulin resistance is associated with several molecular alterations in adipose tissue, such as increased macrophage infiltration, especially in the visceral depots, metabolic adaptations, ER stress, and mitochondrial dysfunction, particularly resulting in elevated protein carbonylation [15]. In the BAT, we investigated the main markers involved in the pathways listed above in a diet-induced obese (DIO) rat. Benefits in reducing oxidative stress and inflammatory processes have been widely reviewed in animal models of obesity treated with bioactive natural compounds, such as flavonoids [16]. Interestingly, adipocyte browning is reported as one of the crucial underlying mechanisms of the anti-obesity properties of extracts from fruits [17]. Red fruits are rich in health-beneficial phytochemicals with antioxidant activities [18]. Furthermore, in 2015, Jeong et al. [19] showed that in high-fat diet (HFD)-induced obese mice given red raspberry extract for 10 weeks, they had reduced weight gain and fat accumulation and an activation of the expression of the thermogenic gene (*UCP-1, PGC-1α*) in BAT [19].

Different studies evaluated the possible benefits of tart cherry consumption in different organs of DIO rats [20,21,22], including WAT [23,24], and a protective effect has been demonstrated when the diet is enriched with antioxidant-rich red-fruit anthocyanins. However, to date, the metabolic modulation and the antioxidant and anti-inflammatory effects of tart cherries (*Prunus cerasus* L.) on BAT in diet-induced obesity have not been examined. The purpose of this study was to investigate whether tart cherry seeds and juice intake decrease HFD-induced alterations in BAT, which may contribute to improving BAT function in obesity.

## 2. Materials and Methods

### 2.1. Animals

Institutional guidelines conforming to the Italian Ministry of Health (protocol number 1610/2013) and associated with guidelines from the European Communities Council Directive were followed to manipulate male Wistar rats. Animals were divided as follows: CHOW rats fed with a standard diet (4RF18, Mucedola, Settimo Milanese, Italy; 2.6 kcal/g), and DIO rats fed with an HFD (45% fat, 35% carbohydrate, 20% protein; D12451, Research Diets, Inc., New Brunswick, NJ, USA; 4.73 kcal/g). The BAT was taken from the same animal model previously described by Micioni Di Bonaventura et al. [22]. After 5 weeks (12 weeks of age) of a hypercaloric diet, the obese phenotype was established. The preparation procedure and the fatty acid composition of seed powder and juice from tart cherries (*Prunus cerasus* L.) were detailed elsewhere [22,23]. DIO rats supplemented with tart cherry seeds (0.1 mg/g/die) were called DS; DS rats supplemented daily with tart cherry juice (containing 1 mg of anthocyanins) were identified with the abbreviation DJS. Body weight and general blood parameters were analyzed and have already been published [20,21,22,23,24]. After 17 weeks of HFD, rats were anesthetized with carbon dioxide and sacrificed. Intrascapular BAT (iBAT) weight was recorded.

### 2.2. Blood Sampling and Analysis

Before the sacrifice, blood was drawn from the tail vein, and the systolic blood pressure was measured afterwards. A total of 800 µL of blood were drawn into tubes containing L-heparin (Sarstedt, Nümbrecht, Germany). Following a 10 min, 3000 rpm centrifugation of the blood samples, the plasma was extracted and kept at −80 °C. Blood parameters (Supplementary Table S1) were evaluated as previously described [20,21,22,23,24]. Leptin concentration was evaluated by the colorimetric method using a specific kit (Rat Leptin ELISA Kit, Abcam ab100773, Cambridge CB2 0AX, UK).

### 2.3. Total Phenolic Compound and Anthocyanin Determinations

Tart cherries were kindly provided by a local company Si.Gi.sas Azienda Agricola (Macerata, MC, Italy). Pulps without the seeds were homogenized at room temperature using a blender for 5 min and subsequently an UltraTurrax^®^ for 1 min. The homogenate was subjected to overnight maceration using ethanol at room temperature to extract the phenolic and organic compounds from the natural matrix of the fruits. After ethanol evaporation, total phenolic compounds and anthocyanins were determined, and all extracts thus obtained were aliquoted and stored at −20 °C until use. Here, the amount of total phenolic compounds in tart cherry samples was determined spectrophotometrically according to the Folin−Ciocalteu method, using gallic acid as a calibration standard [25]. Aliquots of 50 µL of the different concentrations of standard and sample were added to 150 µL of freshly prepared Folin–Ciocalteu Reagent (FCR) (1:4 *v*/*v* in distilled water). After 10 min at 37 °C, 50 µL of a saturated solution of sodium carbonate was added. The absorbance of each solution was revealed at 765 nm. The total amount of phenolics was calculated as mg Gallic Acid Equivalent (GAE)/g sample. Total monomeric anthocyanin content was measured by the pH differential method [26]. Total anthocyanins were expressed as cyanidin-3-glucoside equivalents per liter using a molar absorptivity of 26,900 L/mol and a molecular weight of 449.2 g/mol. In the current study, the total amount of phenolic compounds was 23 ± 1 GAE/g sample, and the total anthocyanins were 0.546 mg/mL. The concentrated juice was standardized so as to give each rat 1 mg of anthocyanins daily for 17 weeks. The preparation of seed powder and juice from tart cherries has already been described in our previous studies [20,21,22,23,24]. As detailed by Cocci [23], the fatty acid composition of ultrasound-assisted extraction (UAE) defatted seeds was distributed in the following percentages: 6.70% palmitic, 0.31% palmitoleic, 4.14% stearic, 43.30% oleic, 44.08% linoleic, 1.05% eicosanoic, and 0.42% eicosenoic.

### 2.4. Morphological Aspects

BAT was removed from both interscapular regions. Tissues were fixed in 4% paraformaldehyde after being dehydrated from graded alcohols and embedded in paraffin. The paraffin tissues were cut using the microtome into sections of tissue 5 µm thick. For the analyses, the sections were deparaffinized by immersing them in xylene and rehydrated using graded alcohols. Subsequently, staining with hematoxylin and eosin (Diapath S.p.A., Martinengo, BG, Italy, Ref. 010263) was performed to evaluate the morphological aspects of the gradual whitening (the presence of white adipocytes, characterized by a large unilocular lipid droplet differently from the brown adipocytes, which possess a multilocular appearance with multiple small lipid droplets) and possible modulations of supplementation-related adipose portions.

### 2.5. Immunohistochemistry

Deparaffinized slides were used for immunohistochemistry (IHC). Heat-mediated antigen retrieval with EDTA buffer pH 8.0 was performed before commencing with the IHC staining protocol. Endogenous peroxidase activity in the adipose organs was blocked by a 30 min incubation with H_2_O_2_, and non-specific binding sites were prevented with normal goat serum and BSA in PBS for 1 h. The sections were incubated with the following primary antibodies diluted in PBS-1% BSA: anti-uncoupling protein-1 (UCP-1) (Thermo Fisher Scientific, Waltham, MA, USA) (1:300), anti-leptin receptor (LepR) (Abcam, Cambridge, UK) (1:200), anti-interleukin-6 (IL-6) (GeneTex, Hsinchu, Taiwan, ROC) (1:500), anti-interleukin-1β (IL-1β) (Novus Biological, Littleton, CO, USA) (1:500), anti-tumor necrosis factor-α (TNF-α) (ABclonal, Seongnam-si, Gyeonggi-do, Korea, 13105) (1:500). The sections were incubated in biotinylated secondary antibody (Bethyl Laboratories, Inc., Montgomery, TX, USA) and then with VECTASTAIN ABC HRP kit, according to the manufacturer’s protocol. 3,3′-diaminobenzidine tetrahydrochloride (DAB) substrate was then applied to the sections. Both of these kits were purchased by Vector Laboratories (Burlingame, CA, USA). Finally, slides were observed under a light microscope, and representative images were captured.

### 2.6. Western Blot

For the Western blots (WB) technique, BAT was lysed in lysis buffer composed of Tris 1M pH 7.4, NaCl 1M, EGTA 10 mM, NaF 100 mM, Na_3_VO_4_ 100 mM, PMSF 100 mM, Deoxycholate 2%, EDTA 100 mM, Triton X100 10%, Glycerol, SDS 10%, Na_4_P_2_O_7_ 0.1M, Protease Inhibitor Cocktail, and distilled H_2_O. After centrifugation, the supernatants were collected, and the protein concentration was determined by a Bradford assay. 40 µg of proteins were separated by 8–14% SDS-PAGE and transferred into a nitrocellulose membrane. Membranes were probed with the primary antibodies diluted in PBS-T at 4 °C overnight. Here, the tested antibodies: anti-UCP-1 (Thermo Fisher Scientific, Waltham, MA, USA) (1:1000), anti-LepR (Abcam, Cambridge, UK) (1:1000), anti-adenosine monophosphate-activated protein kinase-α (AMPK-α) (Abcam, Cambridge, UK) (1:1000), anti-phospho-AMPK-α (Cell Signaling Technology, Inc., Danvers, MA, USA) (1:000), anti-IL-6 (Santa Cruz Biotechnology, Inc., Dallas, TX, USA) (1:200), anti-IL-1β (Novus Biologicals, Littleton, CO, USA) (1:1000), anti-TNF-α (ABclonal, Seongnam-si, Gyeonggi-do, Korea, 13105) (1:500), anti-glucose-regulated protein-94 (GRP-94) (Cell Signaling Technology, Inc., Danvers, MA, USA) (1:1000), anti-caspase-3 (Cell Signaling Technology, Inc., Danvers, MA, USA) (1:1000), anti-4-hydroxynonenal (4-HNE) (Santa Cruz Biotechnology, Inc., Dallas, TX, USA) (1:500).

After incubation with horseradish-peroxidase (HRP)-conjugated secondary antibodies (Bethyl Laboratories, Inc., Montgomery, TX, USA), followed by the enhanced chemiluminescence (ECL) method, protein signals were measured by densitometry with Nikon Imaging Software (NIS Elements AR 4.30.02) (Nikon, Florence, Italy) using β-actin (A2228, Sigma-Aldrich Co., St. Louis, MO, USA) as a loading control.

In addition, the protein carbonyl levels were analyzed with the OxyBlot Protein Oxidation Detection Kit (S7150, Merk-Millipore, Burlington, MA, USA). According to the manufacturer’s instructions, because of the oxidative modification of proteins, carbonyl groups were introduced into protein side chains that were derivatized into 2,4-dinitrophenylhydrazone. The derivatized protein samples were separated by WB. The membrane was incubated with the primary antibody, followed by incubation with a horseradish peroxidase conjugate secondary antibody directed against the primary antibody.

### 2.7. Gene Expression Analysis

The mRNA expression levels of the *PR domain containing 16* (*PRDM16*), *PGC-1α*, *UCP-1*, *leptin*, *adiponectin*, and *18s rRNA* (as an internal control) in BAT were determined by a SYBR green-based real-time polymerase chain reaction (qRT-PCR) with gene-specific primer pairs (Table 1). Total RNA was extracted from samples using QIAzol Lysis Reagent, according to the manufacturer’s instructions (Qiagen, Hilden, Germany). The extracted RNA was quantified using a Qubit™ 4 Fluorometer (Thermo Fisher Scientific, Waltham, MA, USA) with the Qubit RNA HS assay kit. The complementary DNA (cDNA) was synthesized from 0.5 μg of RNA by using the 5X All-In-One RT MasterMix (with AccuRT Genomic DNA Removal Kit) following manufacturer’s guidelines (abm). qRT-PCR was carried out in an ABI 7300 Real-Time PCR System (Applied Biosystems Inc., Waltham, MA, USA). Briefly, the reaction included 2X BlasTaq™ qPCR MasterMix (abm), forward and reverse primers (10 μmol L^−1^), 2 μL cDNA template, and DEPC-treated H_2_O. Thermo-cycling was at 95 °C for 3 min, followed by 40 cycles of 15 s at 95 °C and 60 s at 60 °C. The amplicon of interest was confirmed with the melting curve analysis. Data were analyzed using the relative 2^−ΔΔct^ method and expressed as normalized fold change corrected for *18s rRNA* with respect to the CHOW group.

### 2.8. Data Analysis

All the results were expressed as means ± standard error of the mean (SEM). For any parameter evaluated, the data were analyzed by analysis of variance (ANOVA), followed by the Bonferroni multiple range tests. A *p* < 0.05 indicates a statistically significant difference.

## 3. Results

### 3.1. Interscapular Brown Adipose Organ Weight

After 17 weeks of HFD feeding, the weight of iBAT was significantly increased in the obese group (DIO: 0.92 ± 0.09 g), compared to the rats fed with a standard diet, used as a control (CHOW: 0.53 ± 0.07 g). Tart cherry supplementation did not significantly modify the weight gain (DS: 0.85 ± 0.08 and DJS: 0.92 ± 0.09 g) (Supplementary Table S1). The data reported in Supplementary Table S1 concerning the general and blood parameters and the WAT weight have already been published [22,24].

### 3.2. Microscopical Analyses of iBAT Morphology

The morphological analysis indicated marked structural differences at the level of the iBAT between control and DIO rats. In the rats fed with the standard diet, the depots were mainly composed of predominantly brown adipocytes.

**Figure 1:**
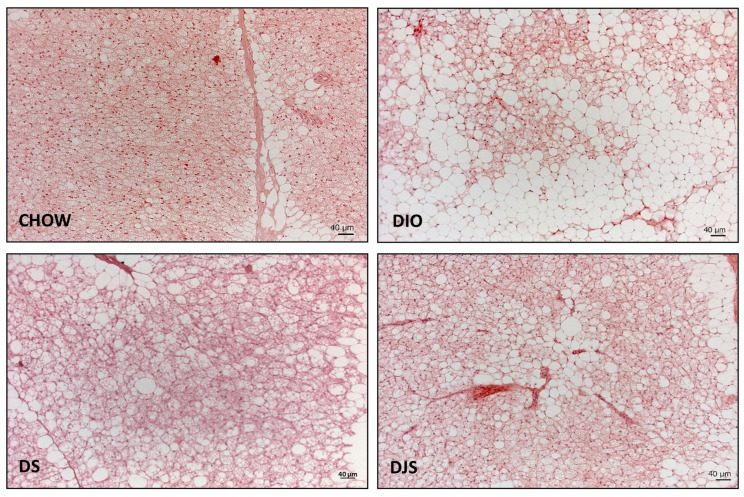
Representative pictures of hematoxylin and eosin staining of interscapular brown adipose tissue. CHOW: rats fed with a standard diet; DIO: rats fed with a high-fat diet; DS: DIO rats supplemented with tart cherry seeds; DJS: DS rats supplemented with tart cherry juice. Magnification 20× Scale bar: 40 μm.

In the DIO group, an expansion of WAT, parallel to the reduction of BAT, can be observed. The HFD added with either tart cherry seeds alone or in combination with tart cherry juice appeared to induce a less severe modification of the iBAT morphology compared to that observed in DIO rats (Figure 1).

### 3.3. Metabolic Assessment of the Whitening Process

**Figure 2:**
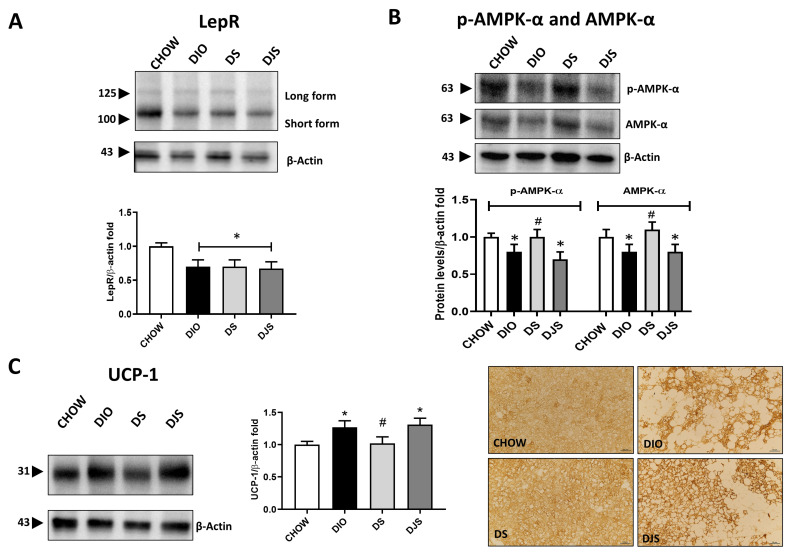
Metabolic evaluation of whitened brown adipose tissue. Western blot analysis of (**A**) leptin receptor isoforms (LepR); (**B**) phospho-adenosine monophosphate-activated protein kinase-α (p-AMPK-α) and adenosine monophosphate-activated protein kinase-α (AMPK-α). Graphs show the densitometric ratios of bands and β-actin expression used to normalize the data. (**C**) Immunoblot of uncoupling protein-1 (UCP-1) and relative graphs that show the trend of the expression of proteins related to β-actin expression were used to normalize the data. Representative pictures of the immunohistochemistry reaction of interscapular brown adipose tissue against UCP-1. CHOW: rats fed with a standard diet; DIO: rats fed with a high-fat diet; DS: DIO rats supplemented with tart cherry seeds; DJS: DS rats supplemented with tart cherry juice. Magnification 20×. Scale bar: 50 μm Data are means ± SEM. * *p* < 0.05 vs. CHOW rats; # *p* < 0.05 vs. DIO rats.

To further evaluate a possible modulation of the iBAT activity and the functional impact of tissue whitening in the different experimental conditions, functional parameters were analyzed. Thus, WB demonstration of leptin receptor isoforms (LepR) in the iBAT showed a relevant decrease in immunoblot, especially of the short isoform at 100 kDa in the DIO group and in groups that received tart cherry supplementation (Figure 2A).

In addition, based on its role in regulating iBAT tissue metabolism, p-AMPK-α and AMPK-α have been tested by WB analysis, which indicated a reduction in the immunoreactivity lane at 63 kDa for rats under HFD and HFD supplemented with tart cherry juice, while an increase in expression at the same level was clear in DS rats (Figure 2B). As shown, both the p-AMPK-α and the AMPK-α total were downregulated in the BAT of DIO rats, and an upregulation was present in the DS group compared to the DIO group (Figure 2B).

The effect of the HFD feeding and the addition of the antioxidant in the diet on the iBAT as well as its impact on the brown-to-white conversion have been further investigated by immunochemistry to visualize the distribution in the tissue of the UCP-1, which was specifically expressed in the functional brown adipocytes. As expected, a diffusely distributed UCP-1 immunoreactivity was produced in control samples. Instead, an obesity-associated reduction of staining distribution was detectable, whose amount appeared to depend on the expansion of the brown-to-white conversion. However, despite such a partial distribution of UCP-1 in iBAT, the intensity of immunoreactivity in the typical multilocular adipocytes was stronger in obese rats than in control ones, as the results of the WB analysis suggest. The distributional patterns of UCP-1 in the iBAT of rats fed with an HFD with tart cherry juice fairly reproduced those described in the DIO rats, while a lower intensity of the UCP-1 staining was observed in the DS group (Figure 2C).

### 3.4. Genetic Analysis of Metabolic Pathways

**Figure 3:**
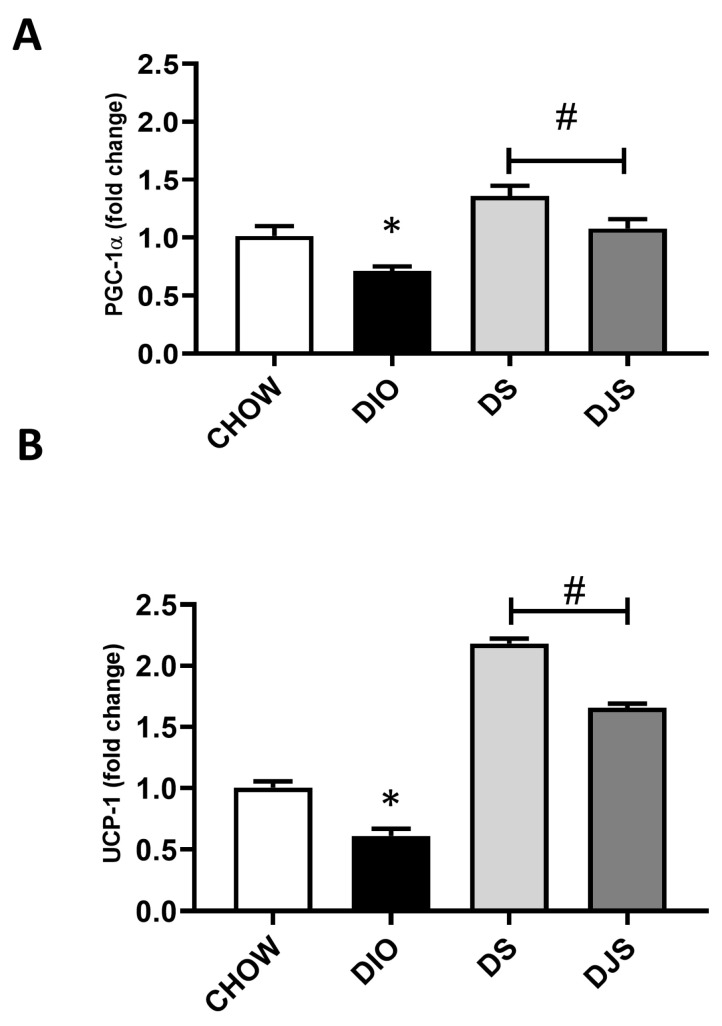
qRT-PCR analysis of the expression of key regulator genes in brown adipocyte metabolic activity. mRNA expression levels of (**A**) *peroxisome proliferator-activated receptor-gamma coactivator 1α (PGC-1α)* and (**B**) *uncoupling protein 1* (*UCP-1*). CHOW: rats fed with a standard diet; DIO: rats fed with a high-fat diet; DS: DIO rats supplemented with tart cherry seeds; DJS: DS rats supplemented with tart cherry juice. Data are means ± SEM. * *p* < 0.05 vs. CHOW rats; # *p* < 0.05 vs. DIO rats.

The qRT-PCR analysis of genes involved in BAT activation was addressed to provide additional data for the assessment of the iBAT activity modulation associated with HFD-induced obesity as well as for a better evaluation of the possible effects of tart cherry supplementation. To this purpose, the expression of transcription factors *PRDM16*, *PGC-1α*, and *UCP-1*, which is the hallmark of thermogenesis [31], has been evaluated (Figure 3).

As expected, in the DIO rats, a significantly reduced expression for *PGC-1α* and *UCP-1* was found (Figure 3A,B). Conversely, the *PRDM16* gene expression was not affected. In addition, tart cherry supplementation resulted in a significant enhanced expression of both *PGC-1α* and *UCP-1* (Figure 3A,B).

**Figure 4:**
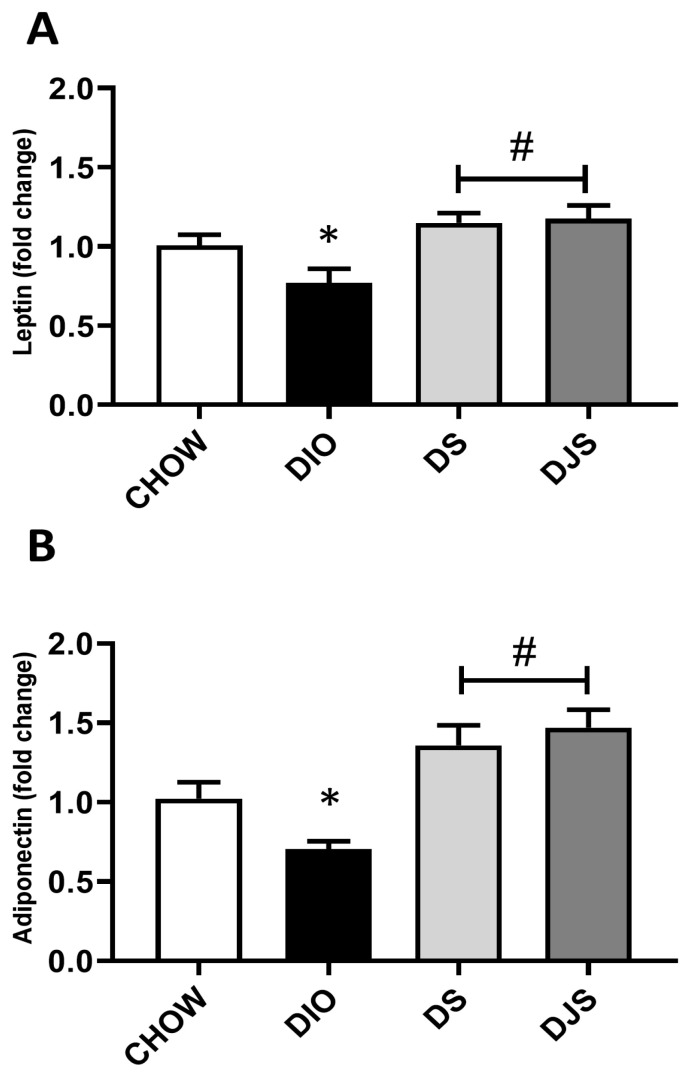
Gene expression of whitened adipocyte-secreted factors. mRNA expression levels of (**A**) *leptin* and (**B**) *adiponectin* in interscapular brown adipose tissue. CHOW: rats fed with a standard diet; DIO: rats fed with a high-fat diet; DS: DIO rats supplemented with tart cherry seeds; DJS: DS rats supplemented with tart cherry juice. Data are means ± SEM. * *p* < 0.05 vs. CHOW rats; # *p* < 0.05 vs. DIO rats.

To evaluate changes in the WAT activity related to the expansion of whitened brown adipocytes in the obese condition, *leptin* and *adiponectin* mRNA were evaluated (Figure 4). For both genes, a significant depletion can be appreciated in the obese groups compared to controls (Figure 4A,B). Moreover, the expression of *leptin* and *adiponectin* genes was found to be significantly upregulated in the tart cherry-supplemented groups (DS and DJS) compared to DIO rats (Figure 4A,B).

### 3.5. Metabolic Stress: Oxidative Stress, Endoplasmatic Reticulum Stress, and Apoptosis

**Figure 5:**
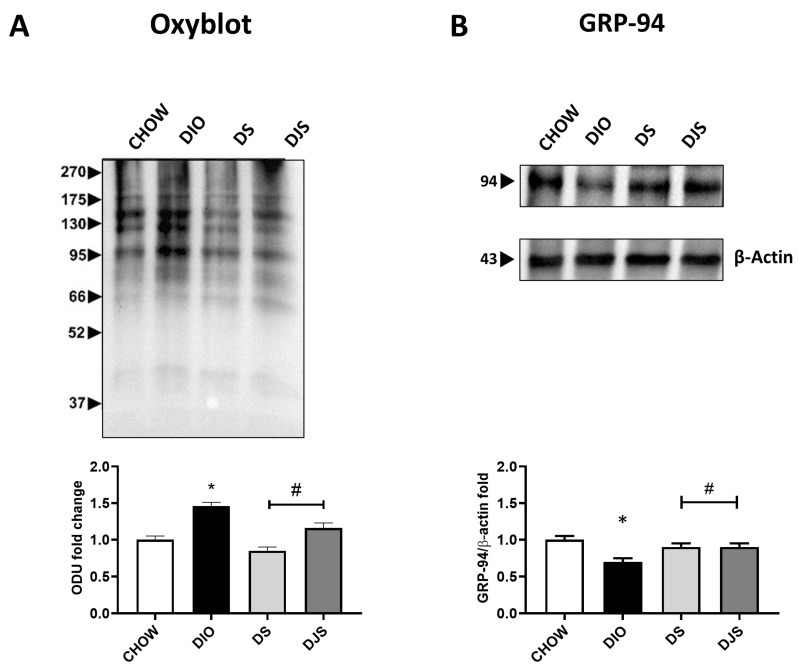
Oxidative and ER stress in brown adipose tissue. Oxyblot in brown adipose samples and the graph demonstrate the measurements of density expressed as optical density units (ODU) (**A**). Adipose tissue lysates were immunoblotted with anti-glucose-regulated protein-94 (GRP-94) (**B**) and relatives’ graphs that indicate the densitometric ratio of band and β-actin expression were used to normalize the data. CHOW: rats fed with a standard diet; DIO: rats fed with a high-fat diet; DS: DIO rats supplemented with tart cherry seeds; DJS: DS rats supplemented with tart cherry juice. Data are means ± SEM. * *p* < 0.05 vs. CHOW rats; # *p* < 0.05 vs. DIO rats.

The quantification of oxyblot assays in adipose tissue homogenates demonstrated an increase in the oxidation state of proteins, respectively in DIO samples compared to control rats. Furthermore, the tart cherries were able to inhibit the oxidative modification of proteins (Figure 5A). The WB analysis for 4-HNE revealed an absence of lipid peroxidation in this specific tissue. Since both dysfunctional hypertrophic adipocytes and the resulting oxidative stress are prone to developing organelle dysfunction, such as ER, we also used GRP94 as a marker of ER stress in iBAT under the different experimental conditions considered here. The results showed a marked depletion of GRP-94 protein expression with its lane investigated at approximately 100 kDa in DIO rats compared with CHOW rats (Figure 5B), suggesting that obesity promotes a UPR impairment. In rats that received the supplemented diet, tart cherry supplementation re-established the normal GRP-94 protein level (Figure 5B). In addition, ER stress is associated with apoptotic cell death [32]; therefore, we investigated apoptosis induction by caspase-3 activation assessment. Our findings showed that no activation by cleavage of caspase-3 was present in any of the different experimental groups.

### 3.6. Inflammation in iBAT

**Figure 6:**
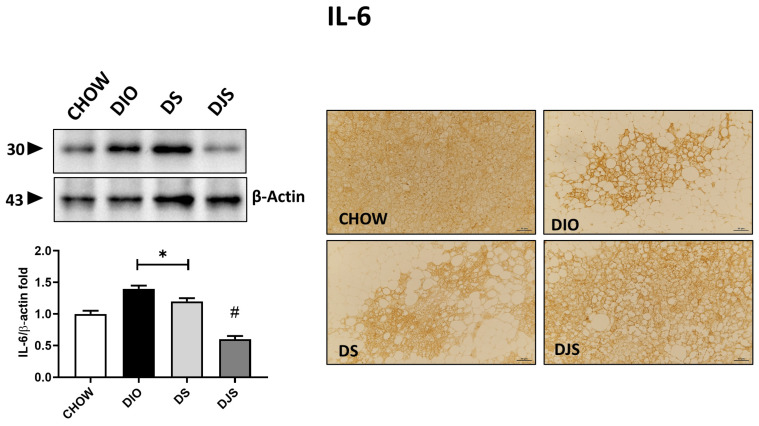
Inflammation in brown adipose tissue. Western blot analysis of interleukin-6 (IL-6). Graph shows the densitometric ratios of bands and β-actin expression used to normalize the data. Representative pictures of the immunohistochemistry reaction of interscapular brown adipose tissue against IL-6. Data are mean ± SEM. * *p* < 0.05 vs. CHOW rats; # *p* < 0.05 vs. DIO rats. CHOW: rats fed with a standard diet; DIO: rats fed with a high-fat diet; DS: DIO rats supplemented with tart cherry seeds; DJS: DS rats supplemented with tart cherry juice. Magnification 20×. Scale bar: 50 μm.

Investigation of the inflammatory status of BAT documented an increase in immune responses in HFD-induced obesity conditions relative to some of the inflammatory markers tested here, such as TNF-α, IL-1β, and IL-6 cytokines (Figure 6). Protein expression of TNF-α and IL-1β, with pro-isoform localized around 31 kDa and mature isoform localized around 17 kDa, was not modulated in this tissue by the HFD diet, our results showed no significant differences among the experimental groups. Different results have been obtained for IL-6, shown at 30 kDa. In fact, a higher intensity of immunostaining has been produced in DIO rats compared to control rats, where very weak to no reactivity was detectable. Rats supplemented with the seeds of the tart cherry showed a significant increase in immunoreactivity compared to the control group, while tart cherry juice added to the HFD attenuated the enhanced production of this cytokine in iBAT, resulting in a weaker immunolabelling concerning the obese rat (Figure 6).

## 4. Discussion

BAT is a thermogenic organ believed to play an important role in human energy homeostasis [33]. When activated, the brown adipocytes in BAT can be an effective energy sink by burning and breaking down excess lipids and glucose. It is now widely accepted that recruitment and activation of BAT can correct dyslipidemia and prevent obesity-related metabolic disorders. Although functional heterogeneity in white and beige adipocytes within a single adipose depot has recently been reported, BAT is still considered a very homogeneous brown adipocyte population [33]. To achieve both safety and multifunctionality, many in vitro and in vivo studies have focused on finding food extracts or natural products that stimulate adipocyte browning by increasing the differentiation of beige adipocytes and improving their functions to combat obesity and related metabolic diseases [34]. Recently, adipocyte browning has been considered one of the fundamental underlying mechanisms of the anti-obesity activities of fruits, tea, and legume extracts [17,35]. Here, we aimed to investigate how iBAT is influenced by HFD-induced obesity in DIO rats and evaluate the possible positive effects of tart cherry supplementation. In accordance with another study [36], we found that DIO promoted BAT whitening, as shown by the morphological changes. The reduction in typical brown adipocytes, in some cases restricted to small areas, and the parallel increased occurrence of white unilocular adipocytes are common features. Besides the typical white adipocytes, white-like brown adipocytes were detectable. These cells largely contribute to the white-like appearance acquired by the tissue in obesity conditions. Tart cherry supplementation to the HFD generally reduced the expansion of the brown-to-white-like conversion in both DS and DJS compared to DIO rats, although no differences in the weight of iBAT were found among the obese groups. Other published works [37,38] report, in agreement with our results, an increase in the weight of the iBAT, or the ratio between the weight of the tissue and the body weight, in conditions of obesity. From the literature, it emerges that this aspect is strictly dependent on the type of diet administered to the animal model, the percentage of fat in the HFD, and the duration of the handling [36].

Such a finding can suggest a possible distinct remodeling of the BAT in response to the tart cherry seed powder and juice. *leptin* and leptin receptor expression were evaluated to analyze the peripheral signaling of caloric intake. In addition, we have already reported *leptin* gene expression modulation in WAT of DIO rats supplemented with the seeds and juice of tart cherries [23]. Moreover, the regulation of energy balance and the thermogenic activity were tested by analyzing AMPK-α and UCP-1 protein expression, respectively. Leptin receptor expression was downregulated in obese animals compared to controls, without differences after supplementation. A decreased concentration of membrane expression of leptin receptors could be associated with impaired leptin sensitivity and, therefore, the pathophysiological state of leptin resistance [39]. Moreover, obesity-related leptin resistance could occur in the hypothalamus, which was named central leptin resistance, while it could also happen in the adipose tissue, liver, and skeletal muscle, which was called peripheral leptin resistance [40]. AMPK-α has been studied for more than two decades as a master regulator of energy balance. It is known that its alteration has been implicated in the onset of obesity and metabolic syndrome. The data regarding the effect of HFD on protein expression of AMPK-α are controversial, with some authors reporting a downregulation [41,42] while others highlighted an increased or no change in protein expression [43,44]. Our results show a significant downregulation of protein expression in the DIO experimental group, compared to the CHOW group, while a possible restoration of AMPK-α is observed in DS but not in DJS groups. There are several possible mechanisms by which HFD might cause the observed decrease in AMPK-α activity. One explanation is that while providing tissues with inadequate amounts of metabolic substrates, such as glucose and free fatty acids, is well known to increase AMPK-α activity, the converse may be true, with an excess of circulating metabolic substrates decreasing AMPK-α activity [45]. Moreover, there is the possibility that HFD might inhibit AMPK-α activities by increasing the ATP/AMP cellular ratio. Another possible construction is the possibility that HFD-induced tissue leptin resistance results in the impairment of intracellular leptin signaling, leading to reduced activation of AMPK-α [46,47]. Our results indicated that only tart cherry seeds restored AMPK-α expression compared to DIO, indicating that oleic and linoleic acids, which mainly compose the tart cherry seed powder [23], are AMPK-α stimulators [48,49,50]. Although future studies are needed to explain the different outcomes in DJS groups, the benefits in an AMPK-α-dependent manner were reported in mice fed an HFD diet supplemented with freeze-dried raspberry [51].

The immunochemical pattern of the UCP-1 showed weak to no UCP-1 immunostaining in the white-like adipocytes of the DIO rats, largely interspersed among strongly reactive brown multilocular adipocytes.

A similar distribution of UCP-1 reactive sites was detected in HFD-fed rats with tart cherry supplementation. However, although individual differences were observed, a common finding is a lower intensity of the UCP-1 staining in typical brown adipocytes and a higher amount of weakly or negative white-like brown adipocytes in DS than in DIO ones. In contrast, in the iBAT of DJS, the increase in UCP-1 cell population could involve the proliferation of brown adipocytes committed to a white-like phenotype. On the other hand, it fits well with the trans-differentiation from beige to white adipocytes proposed for the subcutaneous WAT expansion upon overnutrition. Such a mechanism might have a positive outcome on HFD as it provides the tissue with new small and mature adipocytes that can store excess fatty acid, although at the expense of the “burning” capacity of the tissue [52].

Analyses of gene and protein expression related to the iBAT thermogenic activity have been addressed to identify differences in the obesity-induced dysfunctional patterns of iBAT in our experimental conditions. As expected, based on the large brown-to-white conversion in obesity, the gene analysis showed a decreased *UCP-1* and related *PGC-1α* expression in DIO rats compared to controls, and the tart cherry treatments restored these changes. An upregulation of thermogenic genes (*PGC-1α, UCP-1*), with respect not only to DIO rats but also to controls, might indicate the activation of a tissue remodeling program distinct from that induced in DIO rats.

Concerning the *UCP-1* results, our data showed that protein and gene expression were not perfectly in agreement, but as reviewed by Fromme and Klingenspor in 2011 [53], there was evidence that expressed protein and its mRNA had a variability that was dependent on the type of diet used in the animal model, the fat percentage of the HFD, or the duration of handling. Moreover, the half-life of the transcript of the *UCP-1* gene and the final protein expressed on mitochondria were subjected to a high turnover rate [53].

Furthermore, as suggested by other authors, the transcriptional induction of adaptive functions in cells can also induce a down-regulation of cellular functions that are not required for the new metabolic condition. As an adaptative mechanism, the cells could reduce the transcriptional levels [54,55,56]. Also, a sort of protein buffering mechanism clearly emerges, according to which stable protein complexes tend to maintain constant protein expression despite changed gene copy numbers and mRNA levels [57,58].

Since in previous studies on WAT, the gene expression of adipocyte-secreted factors *leptin* and *adiponectin* was modulated during an HFD and tart cherry supplementation [23], here they were investigated to evaluate changes in metabolic activity. Our results showed a significant reduction in the transcripts of both *leptin and adiponectin* genes in the iBAT genes of DIO rats; these levels were markedly lower than in WAT [23]. On the contrary, increased levels of transcripts were detected in supplemented groups, which suggests a restored leptin-dependent mechanism of energy expenditure via thermogenesis, as suggested by Barrios in 2021 [59].

As reported in the literature, leptin expression in BAT has been somewhat controversial, even more so in obese conditions [9,10,60,61,62,63]. As expected, we found an increase in leptin concentration in the plasma of the obese model. To date, there are no publications regarding the concomitant evaluation of UCP-1 and leptin expression in the BAT of rats fed specifically a HFD diet supplemented with tart cherries seeds and juice. mRNA *leptin* is expressed in WAT and also in BAT; however, its expression in brown fat is decidedly lower than in white fat. It was proposed that *leptin* expression in BAT is due to the presence of white adipocytes that reside within BAT and that brown adipocytes do not express *leptin* or express *leptin* mRNA at very low levels both in rat brown fat as well as mouse brown fat. In addition, it was found through an immunohistochemical approach that UCP-positive cells with typical brown mitochondria do not express leptin [9]. Moreover, we speculated that these are adipocytes that have undergone a whitening process and would not be perfectly ‘functioning’. Furthermore, BAT has been shown to have high plasticity and heterogeneity. Therefore, it is composed of different cell populations with various degrees of whitening and, consequently, with different metabolic structures. However, the molecular mechanisms of other regulatory factors mediating the expression of leptin in the BAT of DIO rats were not the focus of the current study.

We speculate that all these aspects mentioned could also be referred to the conditions studied in our work, in which brown adipocytes subjected to stress induced by a high-caloric diet undergo a whitening process, which induces the cells to reduce the transcription of genes such as *UCP-1*, relating to the metabolic structure of the brown adipocyte.

Consistent with the increased cellular size of these white-like unilocular elements, the tissue displays an imbalance between the production and inactivation of ROS, which contributes to cellular dysfunction [52]. In our results, oxidative stress significantly increases in obese conditions and then re-establishes at levels closer to control in tart cherry-supplemented rats. This would attest to the beneficial effects of both the seeds and the juice of tart cherries [64] already seen in the liver and heart of DIO rats [20,21]. In iBAT, our results suggest no HFD-related modulation of metabolites derived from lipid peroxidation, as reported by [15]. Several findings demonstrated that ER stress develops in the BAT of HFD-individuals, playing an important role in the whitening process [65]. During ER stress, the UPR pathways are actuated to replace the unfolded polypeptides and rebuild the ER physiological balance. In accordance with these findings, we report a marked down-regulation of the expression levels of GRP-94, a chaperone belonging to the Hsp90 family, in DIO rats, which suggests a deteriorated UPR pathway. Interestingly, supplementation with tart cherries on a diet seems to report GRP-94 protein levels near control expression. Moreover, we investigated the possible interconnection between oxidative stress and apoptosis. The protein expression of procaspase-3 and cleaved caspase-3 active isoforms was evaluated. Our results showed that no cleavage of caspase-3 was present in any of the experimental groups, suggesting that BAT remodeling induced by diet and supplementation is not associated with cell death.

Pro-inflammatory cytokine expression was analyzed to investigate the presence of lymphocytic infiltrates and the subsequent enhancement of the pro-inflammatory microenvironment. Here, TNF-α and IL-1β protein expression were evaluated by WB and IHC, and no statistically significant modulation has been reported among the different experimental groups. Compared to WAT, BAT depots are less susceptible to developing local inflammation in response to obesity [66]. In addition, considering the endocrine secretion capacity of WAT in regard to adipokines, BAT was considered poorly secretory. Today, it is well known that BAT exerts an important endocrine function through the secretion of so-called batokines. Among these, IL-6 stands out, which appears to represent a stress-inducible endocrine factor released by BAT [67]. This explains our WB and IHC results, which demonstrated a significant enhancement of IL-6 expression in the DIO and DS groups compared to CHOW rats. In contrast, a restored level of IL-6 was found in the groups that received tart cherry juice fruits, confirming the positive role of these supplements against obesity-induced inflammation [68,69]. Seymour and co-workers [70] reported, in obesity-prone rats fed with an HFD, that the intake of tart cherries reduced retroperitoneal IL-6 and TNF-α mRNA expression, Nuclear factor kappa B (NF-κB) activity, and plasma IL-6 and TNF-α concentrations. These results were consistent with our previous data in heart [21] and in WAT [24]. Also, in the current manuscript, BAT demonstrated that among all inflammatory biomarkers, only some cytokines were significantly modulated in obese rats following tart cherry seed and juice consumption.

Finally, the differences we found between the DS and DJS groups in the BAT’s protein expression as well as in other organs [20,21,22,23,24] can be attributed to the fatty acid composition (e.g., oleic and linoleic acids) of seeds or anthocyanin-enriched tart cherry juice. The biological efficiency of tart cherries may be due to phytochemical interactions that achieve complementary properties. Therefore, combinations of cherry secondary metabolites or whole cherry products may have biological activities that are different from individual components. Such a cooperative result denotes cases in which mixtures of bioactive substances exert effects on target sites that are greater than the sum of the components taken separately [64,71,72,73]. In our study, the anthocyanins of tart cherries appeared effective in triggering molecular changes in BAT, impacting the systemic metabolic homeostasis altered by obesity.

About the effects of tart cherry supplementation on the animal model studied, we discussed some interesting works [74,75,76] in which the authors describe the positive effects on BAT activity in similar animal models, including an increase in UCP-1 and the reduction of inflammatory markers, including IL-6. We speculate that a combination of fatty acids and anthocyanins present in our tart cherry seeds powder, and juice may stimulate the browning process observed in our work.

These results are in line with those obtained in our previous papers about the tart cherries’ ability to counteract obesity-induced alterations in WAT [23,24]. The effects of anthocyanin supplementation remain controversial in mice fed an obesogenic, high-fat diet. Indeed, further investigation into the cellular mechanisms regulated by tart cherry supplementation is required, especially to support consistent clinical outcomes.

## 5. Conclusions

In conclusion, in BAT depots of the interscapular organ, morphological investigation and expression analyses of key genes and proteins related to metabolic activity revealed obesity-induced tissue dysfunction characterized by the expansion of WAT versus BAT. These findings demonstrate the occurrence of an adipocyte whitening program, mainly because of the conversion of brown adipocytes to white-like unilocular cells, which play a crucial role in regulating adipose tissue remodeling under conditions of nutrient excess. Moreover, tart cherry supplementation seems to restore a low level of protein carbonylation, enhance the UPR cellular response, and improve the metabolic pathway of brown adipocytes, stimulating UCP-1 and AMPK-α involved, respectively, in thermogenesis and energy sensitivity.

## Figures and Tables

**Table 1:** List of qRT-PCR primer sequences.

Gene	Primer Sequences	References	Accession Number
*Adiponectin*	GAGAAGGGAGACGCAGGTGT GCTGAATGCTGAGTGATACATGTAAG	[27]	XM_039087986
*Leptin*	GACATTTCACACAGGCAGTCG GCAAGCTGGTGAGGATCTGT	[27]	XM_032905243
*UCP-1*	GCCATCTGCATGGGATCAAACC TCGTCCCTTTCCAAAGTGTTGAC	[28]	NM_009463
*PGC-1α*	CTCCATGCCTGACGGCACCC GCAGGGACGTCTTTGTGGCT	[29]	XM_032916065
*PRDM16*	CACGGTGAAGCCATTCATATGCG AGGTTGGAGAACTGCGTGTAGG	[28]	XM_006539171
*18s rRNA*	GCCGCTAGAGGTGAAATTCTTG CATTCTTGGCAAATGCTTTCG	[30]	NR_046237

## Data Availability

The data presented in this study are available on request from the corresponding author.

## Supplementary Materials

The following supporting information can be downloaded at: https://www.mdpi.com/article/10.3390/antiox13040388/s1, Table S1: General and blood parameters and adipose tissue weight at the end of supplementation.
