# Supplementary material for: Dysfunction of the Brown Adipose Organ in HFD-Obese Rats and Effect of Tart Cherry Supplementation
Source: Antioxidants (Basel). 2024 Mar 23;13(4):388. doi: 10.3390/antiox13040388 (PMC11047636; doi:10.3390/antiox13040388)
Supplement: Supplementary file 1 [file antioxidants-13-00388-s001.zip › antioxidants-2889970-supplementary.pdf]

**Supplementary Table S1.**
General and blood parameters and adipose tissue weight at the end of supplementation.

| | **CHOW** | **DIO** | **DS** | **DJS** |
|---|---|---|---|---|
| **General parameters** | | | | |
| Body Weight (g) | 557.0 ± 10.7 | 682.8 ± 17** | 683.1 ± 29.7** | 689 ± 20.8** |
| Food Intake (kcal) | 75.7 ± 2.3 | 93.0 ± 3.3* | 91.6 ± 7* | 88.0 ± 2.6* |
| **Adipose tissue weight** | | | | |
| Perigonadal white adipose tissue weight (g) | 9.5 ± 1.1 | 19.6 ± 1.4* | 16.9 ± 1.1* | 20.4 ± 2.5* |
| Retroperitoneal white adipose tissue weight (g) | 8.8 ± 0.9 | 29.4 ± 3.8* | 25.1 ± 11.8* | 29.4 ± 3.1* |
| Interscapular brown adipose tissue weight (g) | 0.53 ± 0.07 | 0.92 ± 0.09* | 0.85 ± 0.08* | 0.92 ± 0.09* |
| **Blood parameters** | | | | |
| Systolic blood pressure (mmHg) | 110.9 ± 6.1 | 140.3 ± 8.1* | 111.4 ± 5.5# | 107.6 ± 6.01# |
| Glycemia (mg/dL) | 91.6 ± 5.1 | 126.8 ± 6.1* | 105.7 ± 3.6# | 111.3 ± 2.4*# |
| Insulin (µg/L) | 0.73 ± 0.05 | 1.06 ± 0.05* | 1.01 ± 0.06* | 1.03 ± 0.06* |
| Cholesterol (mg/dL) | 76.1 ± 3.3 | 75.6 ± 4.1 | 69.8 ± 5.6 | 77.6 ± 4.4 |
| Triglycerides (mg/dL) | 76.6 ± 10.4 | 84.3 ± 13.9 | 42.9 ± 3.6*# | 49.8 ± 1.9*# |
| Leptin concentration (pg/mL) | 3449.7 ± 165.6 | 9159.2 ± 110.1* | 8362.6 ± 184.5*# | 7572.0 ± 65.9*# |

CHOW: rats fed with standard diet; DIO: rats fed with high-fat diet; DS: DIO rats supplemented with tart cherry seeds; DJS: DS rats supplemented with tart cherry juice. Data are the means ± S.E.M. * $p < 0.05$, ** $p < 0.01$ vs. CHOW rats; # $p < 0.05$ vs. DIO rats.

Modified from previous articles:


- Micioni Di Bonaventura, M.V.; Martinelli, I.; Moruzzi, M.; Micioni Di Bonaventura, E.; Giusepponi, M.E.; Polidori, C.; Lupidi, G.; Tayebati, S.K.; Amenta, F.; Cifani, C.; Tomassoni, D. Brain alterations in high fat diet induced obesity: effects of tart cherry seeds and juice. *Nutrients.* 2020, *12*, 623. doi: 10.3390/nu12030623

- Moruzzi, M.; Klöting, N.; Blüher, M.; Martinelli, I.; Tayebati, S. K.; Gabrielli, M. G.; Roy, P.; Micioni Di Bonaventura, M. V.; Cifani, C.; Lupidi, G.; Amenta, F.; Tomassoni, D. Tart Cherry Juice and Seeds Affect Pro-Inflammatory Markers in Visceral Adipose Tissue of High-Fat Diet Obese Rats. *Molecules*2021, *26*, 1403. doi.org/10.3390/molecules26051403.
